# The proportion of endometrial cancers associated with Lynch syndrome: a systematic review of the literature and meta-analysis

**DOI:** 10.1038/s41436-019-0536-8

**Published:** 2019-05-14

**Authors:** N. A. J. Ryan, M. A. Glaire, D. Blake, M. Cabrera-Dandy, D. G. Evans, E. J. Crosbie

**Affiliations:** 1 Division of Cancer Sciences, Faculty of Biology, Medicine and Health, University of Manchester, St Mary’s Hospital, Manchester, UK; 2 Division of Evolution and Genomic Medicine, University of Manchester, St Mary’s Hospital, Manchester, UK; 3 Tumor Genomics and Immunology Group, The Oxford Centre for Cancer Gene Research, Wellcome Trust Centre for Human Genetics, University of Oxford, Oxford, UK; 4 Department of Obstetrics and Gynaecology, St James’s University Hospital, The Leeds Teaching Hospitals NHS Trust, Leeds, UK; 5 Lancashire Hospitals NHS Trust, Royal Blackburn Hospital, Blackburn, UK; 6 Manchester Centre for Genomic Medicine, Manchester University NHS Foundation Trust, Manchester Academic Health Science Centre, Manchester, UK; 7 Department of Obstetrics and Gynaecology, Manchester University NHS Foundation Trust, Manchester Academic Health Science Centre, Manchester, UK

**Keywords:** systematic review, Lynch syndrome, endometrial cancer, mismatch repair (MMR) immunohistochemistry, microsatellite instability (MSI)

## Abstract

**Purpose:**

Endometrial cancer (EC) is often the sentinel cancer in women with Lynch syndrome (LS). However, efforts to implement universal LS screening in EC patients have been hampered by a lack of evidence detailing the proportion of EC patients that would be expected to screen positive for LS.

**Methods:**

Studies were identified by electronic searches of Medline, Embase, Cochrane CENTRAL and Web of Science. Proportions of test positivity were calculated by random and fixed-effects meta-analysis models. *I*^2^ score was used to assess heterogeneity across studies.

**Results:**

Fifty-three studies, including 12,633 EC patients, met the inclusion criteria. The overall proportion of endometrial tumors with microsatellite instability or mismatch repair (MMR) deficiency by immunohistochemistry (IHC) was 0.27 (95% confidence interval [CI] 0.25–0.28, *I*^2^: 71%) and 0.26 (95% CI 0.25–0.27, *I*^2^: 88%), respectively. Of those women with abnormal tumor testing, 0.29 (95% CI 0.25–0.33, *I*^2^: 83%) had LS-associated pathogenic variants on germline testing; therefore around 3% of ECs can be attributed to LS. Preselection of EC cases did increase the proportion of germline LS diagnoses.

**Conclusion:**

The current study suggests that prevalence of LS in EC patients is approximately 3%, similar to that of colorectal cancer patients; therefore our data support the implementation of universal EC screening for LS.

## INTRODUCTION

Lynch syndrome (LS) is an autosomal dominant cancer predisposition syndrome. Those affected most commonly inherit an inactivating variant in one of the four mismatch repair system (MMR) genes: *MLH1*, *MSH2*, *MSH6*, or *PMS2*. This highly conserved system is responsible for correcting insertion and deletion errors that occur during genomic replication.^1^ Loss of MMR functioning, termed MMR deficiency (MMRd), leads to microsatellite instability (MSI),^2^ a hypermutated phenotype, and increased cancer susceptibility. LS patients are at an increased risk for a number of different malignancies, but most commonly develop colorectal and endometrial cancer.^3,4^ As a result, patients with LS have a decreased life expectancy compared with nonaffected individuals.^5^

Diagnosing LS in endometrial cancer (EC) patients is an important step in clinical management. It allows for cascade testing to diagnose family members who may also have the disease.^6^ Furthermore, timely LS diagnosis allows for the initiation of lifestyle modification such as weight loss, chemoprophylaxis, and cancer site surveillance to prevent the development of further LS-related malignancies, most notably colorectal cancer (CRC).^4,7^ Annual colonoscopy has been shown to improve overall survival in LS patients through the detection and removal of adenomatous polyps.^4^ There is a growing drive for universal screening of CRC patients for LS.^8–10^ Indeed, the National Institute of Health and Care Excellence (NICE) in the United Kingdom has recently introduced a LS screening pathway for all CRC patients, alongside numerous institutions in the United States.^11^ LS screening pathways utilize tumor-based testing (immunohistochemistry [IHC] for MMR protein loss, MSI testing or *MLH1* promoter methylation testing) to triage cases to undergo germline testing to identify a pathogenic variant in one of the MMR genes.

Universal screening of EC patients for LS has been recommended by numerous experts and specialist societies.^12^ Such practice has already been adopted in several cancer centers across the world.^13–15^ Proponents suggest a similar proportion of EC is related to LS as seen in CRC. Furthermore, there is evidence that EC is often the sentinel cancer in women with LS.^16^ Therefore, a diagnosis of LS at the time of EC diagnosis could afford earlier CRC surveillance and achieve greater survival benefit. However, the true proportion of EC associated with LS remains unclear. Published proportions vary greatly, with estimates ranging from 1% to around 10%.^17,18^ Such variation in estimates is in part due to variable testing strategies employed across different studies.

In this systematic review we sought to provide accurate data estimating the outcomes of testing for LS in EC patients. Specifically, we asked what proportion of EC patients would be expected to be put forward for definitive germline testing following initial tumor-based tests (namely IHC, MSI with or without *MLH1* promoter methylation analysis), and secondly, what proportion of these would be confirmed Lynch syndrome by next-generation sequencing (NGS). The results of this study may be of benefit in informing the planning and implementation of universal LS screening in EC patients.

## MATERIALS AND METHODS

### Search strategy and study identification

A systematic literature search devised by a specialist librarian, following PRISMA guidelines,^19^ was undertaken. Medline, Embase, Cochrane CENTRAL, and Web of Science were searched. The gray literature and nonelectronic literature were not included. Search terms were “colorectal neoplasms, hereditary nonpolyposis” and “endometrial cancer” with associated Medical Subject Headings (MeSH). In addition, a secondary search was conducted using “Lynch syndrome” as a multipurpose term and “endometrial cancer” as a MeSH term. The search included all studies from source commencement to the end of July 2018. Citation searching was utilized to augment the initial results.

Studies found to have inconsistent results were excluded after unanimous review and agreement between all authors. Assessment of bias analysis was conducted by three reviewers (N.A.J.R., D.B., and M.C.D.) independently using Review Manager (RevMan) (Version 5.3. Copenhagen: The Nordic Cochrane Centre, The Cochrane Collaboration, 2014). Disagreements were resolved by either unanimous agreement after rereview or by the decision of the senior author (E.J.C.).

### Selection criteria

The protocol for this systematic review was preregistered with the PROSPERO database registration (ID: CRD42017081707) and has been published.^20^ Only studies investigating LS in an EC population were included. Initial searches were limited by English language, human adults (>18 years), and female subjects. Only studies that used either direct germline analysis for pathogenic variants of MMR genes or proxy tumor-based molecular diagnostic methods (IHC, MSI with or without *MLH1* promoter hypermethylation), or any combination of these were included. Microsatellite instability-high (MSI-H) was defined, where possible, as involving ≥30% of the included microsatellite markers. An IHC positive result was taken as loss of expression of one of the MMR proteins. Pathogenic variants of MMR genes were defined as per the authors’ analysis. To avoid double counting data, authors of more than one study were contacted for clarification and/or registry analysis was crosschecked. Where there was overlapping data, the larger study was included and the smaller excluded. Only articles that contributed at least 15 participants were included.

### Data extraction

The results from the initial search were combined. The titles and abstracts were collated in a spreadsheet template downloaded from http://libguides.sph.uth.tmc.edu/excel_workbook_home. This is available from the authors on request. Duplicates were removed with the use of Endnote X7 (Thompson Reuters, New York, NY). All titles and abstracts where initially screened independently by three authors (N.A.J.R., D.B., and M.C.D.). Conflicts were resolved by unanimous agreement between the three observers. Where unanimous agreement could not be reached a senior author (E.J.C.) made the final decision. Those studies identified as meeting the inclusion criteria underwent full article review and data extraction. Those excluded at full manuscript review are detailed in supplementary materials appendix 1.

Baseline data extracted included type of study, selection criteria, number of participants, country of origin, demographic data, type of initial screening method for LS, gold standard test, pathogenic variant distribution, and pathology distribution.

### Statistical analysis

The primary outcome measure was the proportion of EC patients who were identified as being likely LS (aberrant MMR IHC expression, MSI-H with or without *MLH1* promoter hypermethylation) or as carrying a germline MMR pathogenic variant. The Freeman–Tukey (a double arcsine transformation) transformed proportions of LS positive EC patients were pooled using the inverse variance heterogeneity model.^21^ To aid interpretation, all results were presented after back transformation to natural proportions. A quantification of heterogeneity across studies was presented as an *I*^2^ score (with *I*^*2*^ score of 25%, 50%, and 75% representing low, moderate, and high levels of heterogeneity respectively).^22^ All statistical analyses were performed R, Version 3.3.1 (https://cran.r-project.org), using the package “meta.”

## RESULTS

### Search results

**Fig. 1:**
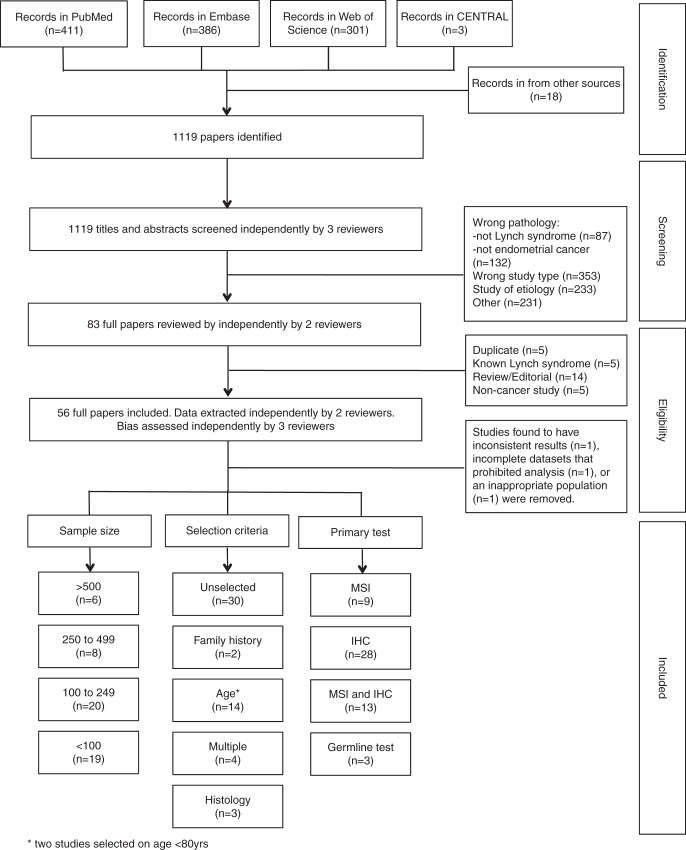
**Flowchart detailing study identification, study selection, and characteristics of included studies.** *IHC* immunohistochemistry, *MSI* microsatellite instability.

The combined search terms yielded 1119 articles. Primary review of titles and abstracts identified 83 articles that warranted full manuscript review. Of these, 56 studies met the inclusion criteria. At data extraction and quality assessment, three studies were removed due to incomplete data (*n* = 1) (ref. ^23^), inconsistent presentation of results (*n* = 1) (ref. ^24^), or an inappropriate population (*n* = 1) (ref. ^25^) (Fig. 1). Bias scores for each of the studies are outlined in supplementary appendix 2.

Fifty-three papers were included for the final analyses.^13–15,17,18,26–73^ These studies included 12,633 participants with EC. The majority of studies were conducted in North America (*n* = 33), with relatively small numbers carried out in Europe (*n* = 6), Southeast Asia (*n* = 7), Australasia (*n* = 4), and South America (*n* = 3). Twenty-three (43%) populations were preselected by age, family history, or other clinical parameters before analysis. Primary testing included MSI (*n* = 9), IHC (*n* = 28), MSI and IHC (*n* = 13), or germline testing (*n* = 3). Studies are summarized in Table 1.

**Table 1:** Summary of included studies

Author	Study year	Country	Selection	Initial tumor screen	Number of participants	Proportion of positive IHC	Proportion of positive MSI	Proportion of negative methylation after positive tumor triage	Proportion of positive germline samples after positive tumor-based triage	Comments
Backes et al.^26^	2009	USA	None	IHC	385	0.12	NA	NA	0.37	IHC results not described by gene; MSI not clearly described
Batte et al.^14^	2014	USA	None	IHC	579	0.07	NA	NA	0.8	Only 36 tumors had MSH6 IHC testing; PMS2 not tested; 20 tumors were MSI-H; 37 were MSI-L
Berends et al.^27^	2003	USA	<50 years	IHC and MSI	58	0.49	0.35	NA	0.09	None
Bruegl et al.^28^	2014	USA	None	IHC	412	0.29	NA	0.11	NA	Only one MS region investigated; only MLH1 and MSH2 IHC carried out; results quoted in the paper are not consistent
Buchanan et al.^29^	2013	Australia	None	IHC	702	0.24	NA	0.09	0.14	Not clear how IHC was applied
Buttin et al.^30^	2004	USA	None	MSI	413	NA	0.27	0.19	NA	None
Catasus et al.^31^	1998	Spain	None	MSI	42	NA	0.29	NA	NA	*PMS2* germline testing not performed; all MSI had GL
Chadwick et al.^32^	2001	USA	None	MSI	74	NA	0.23	NA	0.12	None
Cook et al.^33^	2013	Canada	<80 years	MSI	480	NA	0.27	NA	NA	Analysis includes 2 known LS carriers
Cossio et al.^34^	2010	Brazil	<50 years of strong FHx	IHC and MSI	30	0.33	0.23	NA	NA	None
Dillon et al.^13^	2017	USA	None	IHC	233	0.26	NA	0.05	0.45	None
Djordjevic et al.^35^	2013	USA	None	IHC	154	0.30	NA	0.14	NA	None
Egoavil et al.^36^	2013	Spain	None	IHC and MSI	173	0.34	0.27	0.14	0.42	89 had unselected GL analysis; 4 GL results not available; those MSI +ve or MLH1 deficient without FHx did not have GL analysis; numbers of IHC MLH1 loss not clear
Ferguson et al.^37^	2014	Canada	None	IHC and MSI	119	0.29	0.23	NA	0.08	A pre universal screening cohort, described in this paper, was not included in this analysis; those who underwent testing not as a result of universal screening were also excluded
Frolova et al.^15^	2015	USA	None	IHC	234	0.22	NA	0.09	0.31	None
Garg et al.^38^	2009	USA	<50 years or LS morphology	IHC	71	0.45	NA	NA	NA	PMS2 not tested
González et al.^39^	2012	Puerto Rico	None	IHC	20	0.25	NA	NA	NA	None
Goodfellow et al.^40^	2015	USA	Endometrioid only	IHC and MSI	1002	0.36	0.30	0.11	0.4	Not all variants detected could be classified as pathogenic
Hampel et al.^41^	2006	USA	None	MSI	543	0.34	0.22	0.06	0.02	Germline pathogenic variants are not described; the PMS2 deficient case on IHC also lacked MSH6 expression
Hartnett et al.^42^	2015	USA	None	IHC	205	0.21	NA	0.03	0.01	Of the 24 tumors tested 5 did not have sufficient material for any MSI; a further 4 had sufficient material for limited analysis
Hewitt et al.^43^	2006	UK	FHx	MSI	17		0.18	NA	NA	None
Joehlin-Price et al.^44^	2014	USA	None	IHC	1054	0.22	NA	NA	NA	None
Kato et al.^45^	2016	Japan	None	IHC	360	0.03	NA	0.02	0.25	Only PMS2 results clearly reported
Kost et al.^66^	2016	USA	<50 years	IHC	83	0.24	NA	0.16		None
Lee et al.^46^	2018	Singapore	<50 years	IHC	315	0.21	NA	NA	NA	None
Leenen et al.^47^	2012	Netherlands	<70 years	IHC and MSI	179	0.23	0.23	0.06	0.7	Only MLH1 and MSH2 on IHC
Lim et al.^48^	1996	USA	None	MSI	28	NA	0.32	NA	NA	Two additional patients with previously known Lynch syndrome added to final results; only 3 germline results are clearly defined
Lin et al.^49^	2016	USA	Mixed	IHC	76	0.22	NA	0.08	0.6	21 of 41 showed MSH2/MSH6 and 10 of 41 showed MLH1/PMS2; however authors also report individual protein loss consisting of 72 in total
Long et al.^66^	2014	China	None	IHC	173	0.24	NA	NA	NA	PMS2 not tested
Lu et al.^50^	2007	USA	<50 years	GL	100	0.34	0.33	0.22	0.09	None
Chu et al.^68^	2015	Hong Kong	<45 years	IHC	67	0.33	0.23	NA		None
Mas-Moya et al.^17^	2015	USA	None	IHC	215	0.33	NA	0.17	0.65	Only those with IHC loss had MSI
Matthews et al.^51^	2008	USA	<50 years	IHC	61	0.34	0.34	NA	NA	MMR IHC done on TMA
McConechy et al.^52^	2015	Canada	None	MSI	89	NA	0.26	NA	NA	MSI results are not clear
Mills et al.^53^	2014	USA	None	IHC and MSI	604	0.25	NA	0.09	0.81	Three phases of testing using different tests and referral criteria; 4 had multiple gene loss on IHC; methylation testing not universally applied and therefore cannot make any sensible deductions from it
Moline et al.^54^	2013	USA	Mixed	IHC or MSI or IHC with Methylation	245	0.25	0.13	NA	0.24	None
Najdawi et al.^69^	2017	Australia	None	IHC	124	0.24	NA	NA	0.33	Only 9 of 11 of the Lynch-like tumors had germline testing
Parc et al.^55^	2000	USA	<52 years	IHC and MSI	62	0.24	0.34	NA	NA	MSH2 and MSH6 IHC outcomes not reported
Pecorino et al.^56^	2017	Italy	<50 years	IHC	41	0.31	0.42	NA	NA	None
Pennington et al.^70^	2013	USA	Serous only	GL	151	NA	NA	NA	0.00	One subject with < 10% serous histology had a *MSH6* pathogenic variant
Rabban et al.^18^	2014	USA	>50 years	IHC	273	0.15	NA	0.04	0.29	None
Resnick et al.^57^	2009	USA	None	IHC	477	0.28	NA	NA	NA	None
Riggi et al.^58^	2016	Argentina	None	IHC	84	0.33	NA	NA	NA	None
Ring et al.^71^	2013	USA	<50 years	IHC	111	0.26	NA	NA	NA	None
Ring et al.^59^	2016	USA	None	IHC or MSI or IHC with methylation	381	NK	NK	NK	0.06	Tumor-based molecular triage was used but the results are not detailed
Rubio et al.^60^	2016	Spain	None	IHC and MSI	94	0.33	0.27	NA	0.15	GL result not clear
Sugawara et al.^61^	2015	Japan	AFP criteria	IHC	182	0.30	NA	0.22	NA	Of all samples 55% not tested
Tan et al.^65^	2013	Australia	<80 years	IHC	246	0.24	NA	NA	NA	None
Walsh et al.^62^	2008	Australia	<50 years	IHC and MSI	146	0.26	0.35	0.18	NA	None
Watkins et al.^73^	2016	USA	None	IHC	242	0.20	NA	0.05	0.4	None
Woo et al.^72^	2014	Malaya	Endometrioid only	IHC	77	0.19	NA	NA	NA	PMS2 not tested
Yoon et al.^63^	2007	Korea	None	IHC and MSI	113	0.23	0.44	0.17	0.04	None
Zauber et al.^64^	2010	USA	None	MSI	213	NA	0.26	0.09	NA	None

*FHx* family history, *GL* germline, *IHC* immunohistochemistry, *LS* Lynch syndrome, *MSI* microsatellite instability, *MSI-H* microsatellite instability high, *MSI-L* microsatellite instability low. *TMA* tissue microarray, *NA* not applicable, *NK* not known, *AFP* age, family and personal history of cancer criteria.

All studies originated from specialist tertiary referral centers or their biobanks. Histological features were reported in 20 papers. Type 1/endometrioid tumors constituted 79.3% of tumors, consistent with the literature.^74^ There were insufficient data to describe the histological breakdown of tumors diagnosed in women who were found to have abnormal tumor triage or LS. From the studies that included age data (*n* = 43), the median age of subjects tested was 59.5 years (interquartile range [IQR:] 53–62).

### Immunohistochemical analysis

**Fig. 2:**
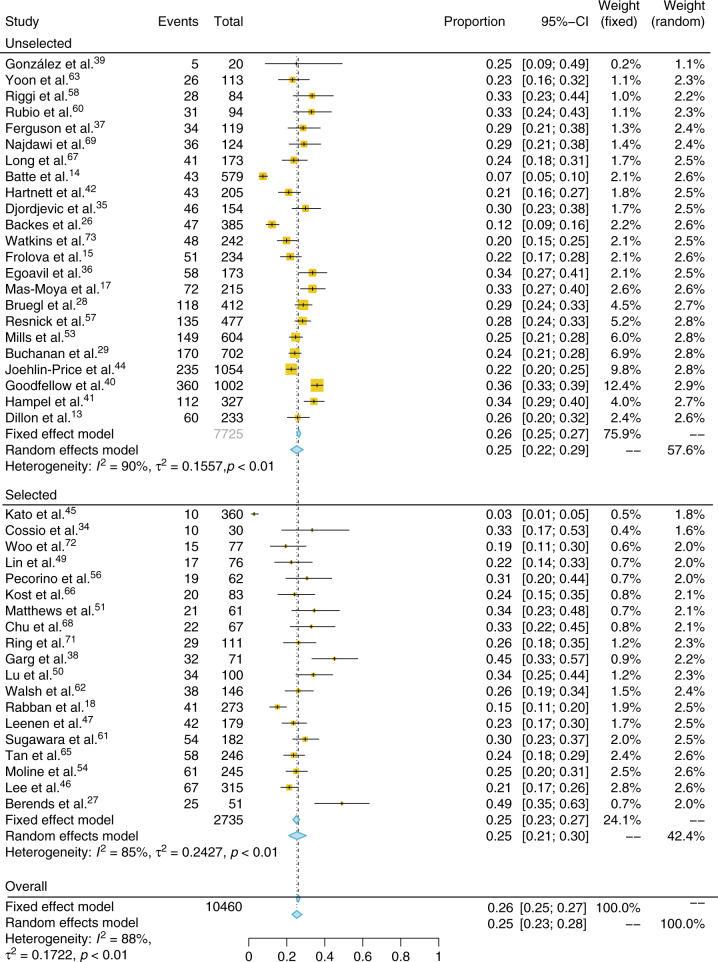
**Forest plot and meta-analysis of the proportion of endometrial tumors with mismatch repair deficiency by immunohistochemistry, including those that did and did not preselect tumors for testing.** *CI* confidence interval.

**Fig. 3:**
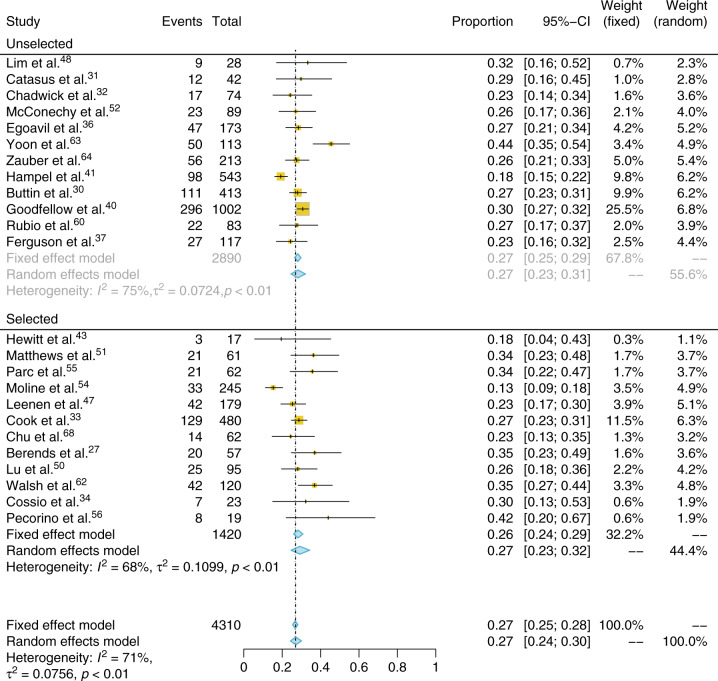
**Forest plot and meta-analysis of the proportion of endometrial tumors showing microsatellite instability, including those that did and did not preselect tumors for testing.** *CI* confidence interval.

In total, 42 papers reported the outcome of IHC analysis. These studies include 10,683 participants, 10,460 with completed IHC analysis. Of these, 2563 (25%) were found to have absent expression of at least one of the MMR proteins on IHC. This represents an overall proportion of 0.26 (confidence interval [CI] 95% 0.25–0.27, *I*^2^: 88%). When preselected populations (cohorts restricted by clinical criteria such as age at EC diagnosis) were excluded, a total of 7725 tumors underwent MMR IHC, of which 1948 (25%) were MMR deficient (Fig. 3). Therefore, the proportion of unselected ECs with MMR deficiency is 0.26 (95% CI 0.25–0.27, *I*^2^: 90%) and 0.25 (95% CI 0.23–0.27, *I*^2^: 85%) in selected EC populations (Fig. 2).

Of note, only a proportion of studies tested for loss in all four of the MMR proteins (23/42, 55%). MLH1-specific outcome data were available for a total of 9306 study participants. Of these, 1635 (18%) were found to be MLH1 protein deficient. When studies that preselected their populations were excluded, the total number of subjects was 7176, of whom 1343 (19%) were MLH1 deficient. Full protein specific data were available for 7385 subjects. Excluding MLH1, deficiencies in MSH6 were the most commonly recorded (*n* = 247/3%), followed by MSH2 (*n* = 211/3%) and finally PMS2 (*n* = 153/2%). As MMR proteins are dimeric, concurrent loss of both proteins within MutSα (MSH2/MSH6) and MutLα (MLH1/PMS2) would be expected. For MSH2, concurrent MSH6 loss was always seen across all studies. For MLH1, PMS2 loss was also seen, with the exception of one case. By contrast, isolated MSH6 was seen in 112 cases and isolated PMS2 loss in 13 cases. In studies with no preselection and with complete protein analysis data there were a total of 6104 study participants. Here the most prevalent protein deficiency was in MSH6 (*n* = 165/3%) followed by MSH2 (*n* = 153/3%) and PMS2 (*n* = 84/1%).

Of 5594 EC with MLH1 IHC and methylation analysis data, 1098 (20%) cases had MLH1 IHC loss. Of these, 963 (88%) were found to be due to somatic hypermethylation of the *MLH1* promoter region. In unselected EC populations with methylation analysis data, there were 4525 participants, the tumors of whom showed MLH1 IHC loss in 960 (21%) cases. Eight hundred forty-three (88%) were as a result of somatic hypermethylation of the *MLH1* promoter region. Therefore the proportion of MLH1 IHC loss not attributed to somatic hypermethylation of MLH1 was 0.11 (95% CI 0.10–0.13, *I*^2^: 78%) (supplementary appendix 4). From these data, there is no significant difference between the proportion of ECs with IHC MLH1 loss and normal methylation analysis according to cohort selection.

### Microsatellite instability analysis

MSI data were available for 4310 tumors, 2580 of which were also tested by IHC. Of these, 1133 (26.3%) were MSI-H. When studies that used preselective criteria were excluded, the total number of participants was 2890, of whom 768 were positive (26.6%) The overall proportion of MSI-H ECs was 0.27 (CI 95% 0.25–0.28, *I*^2^: 71%). This was 0.27 (CI 95% 0.25–0.29, *I*^2^: 75%) and 0.26 (CI 95% 0.24–0.29, *I*^2^: 68%) for unselected and preselected populations, respectively (Fig. 3). The results of *MLH1* methylation analysis in conjunction with MSI testing are shown in supplementary appendix 4.

There was no significant difference between the proportion of positive test results if IHC or MSI was used as the initial tumor triage (0.25 vs. 0.27 *p* value = 0.5 [Student's *t* test]). Analysis of MSI proportions pre-2011 vs. post-2011 did not find a significant difference (*t* test *p* value = 0.11).

### Germline analysis

In total, 23 studies used some form of germline analysis to establish a diagnosis of LS. There was a wide degree of variation in the completeness of germline testing, with few studies (*n* = 9) testing all those they intended to. Furthermore, 12 studies that used a tumor-based triage test did not perform *MLH1* promoter methylation testing. Two studies failed to report the outcome of their *MLH1* methylation testing.^14,54^ The majority of these studies used another means of excluding a proportion of their MLH1 IHC positive results, for example a negative family history. Therefore, the population that went on to have definitive germline analysis is heterogeneous.

**Fig. 4:**
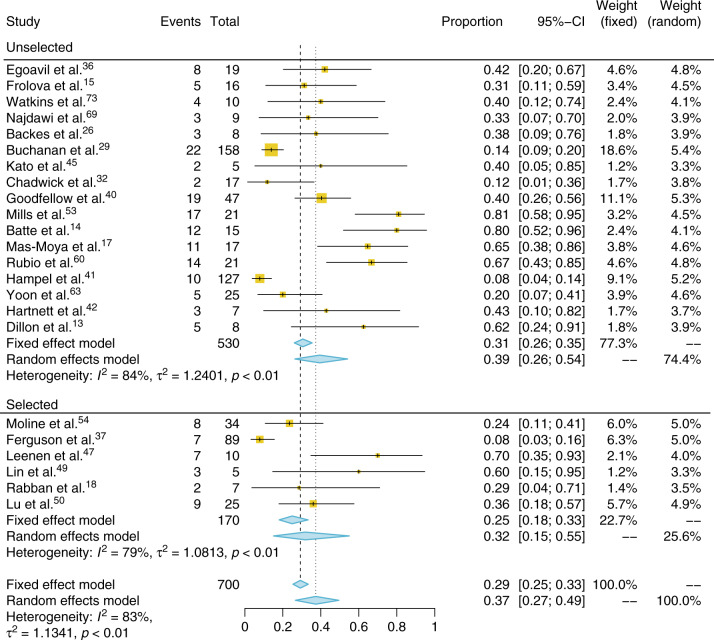
**Forest plot and meta-analysis of the proportion of pathogenic variants in mismatch repairs (MMR) genes found in women with endometrial tumors showing mismatch repair deficiency and/or microsatellite instability, including studies that did and did not preselect women for testing.** *CI* confidence interval.

In total, 14,770 tumors underwent tumor-based triage with IHC (*n* = 10,460) and/or MSI (*n* = 4310). Concurrent testing with both IHC and MSI was sufficiently reported in ten studies^16,27,34,36,37,40,47,51,63,68^ and enabled removal of duplicates with positive concordant IHC and MSI data. Of the remaining 14,293 tumors, 1133 were MSI-H and 2563 had aberrant IHC. Studies that reported MLH1-specific IHC and *MLH1* promoter methylation tumor outcomes (*n* = 5594) allowed further triage by removing tumors with likely somatic *MLH1* loss. In total, 1005 women were eligible for and 700 women underwent germline testing for Lynch syndrome following tumor-based triage. A total of 181 (26%) were positive. When studies that preselected their population were excluded, the combined population was 5882, of whom, upon removal of methylated results, 821 (14%) were Lynch-like on the basis of their tumor analyses and therefore should have undergone germline testing. A total of 511 (62%) underwent testing and 150 (29%) were positive. This represents an overall proportion of LS-related pathogenic variants in those with positive tumor triage of 0.29 (CI 95% 0.25–0.33, *I*^2^: 83%). The same proportion in unselected cases was 0.31 (CI 95% 0.26–0.35, *I*^2^: 84%) and for selected 0.25 (CI 95% 0.18–0.33, *I*^2^: 79%) (Fig. 4). Therefore, around 3% of ECs can be attributed to LS. The gene breakdown from NGS is shown in supplementary appendix 3.

Four studies, which examined unselected populations of EC, had complete germline testing of cases suggestive of LS on the basis of their tumor analysis. Focusing on these studies, 180 tumors were suggestive of LS of which 32 were found to have a pathological variant in one of the MMR genes. This represents a proportion of 0.21 (CI 95% 0.15–0.30, *I*^2^: 91%). This is significantly fewer than reported in studies with incomplete germline testing (*p* = 0.001) and suggests an overreporting of pathological variants in studies that fail to analyze all samples suggestive of LS from tumor triage. Only three studies did direct germline analysis without tumor triage. These studies were all highly selective, with Pennington and colleagues testing only tumors of a serous histology, which is not commonly associated with LS.^75^ Of 590 cancers tested in these three studies, 27 (4.6%) were found to have a germline pathological variant. If the data from Pennington are removed, 6% of ECs were found to have pathogenic variants associated with LS. These data are shown in supplementary appendix 5. Subgroup analysis of pre-2011 vs. post-2011 proportions of germline pathogenic variants found after tumor triage was not significant (*t* test *p* value = 0.14).

Further subgroup analysis was carried out to explore the relationship between potential confounding factors; these are shown in supplementary appendix 5. Of note, those studies that did not use a priori tumor-based triage, using instead direct germline sequencing of all samples, found a higher proportion of LS carriers (0.06); however, the number of studies is limited (*n* = 3). In addition, limiting testing to individuals less than 50 years yielded higher levels of MSI (0.31) and aberrant IHC (0.28).

## DISCUSSION

Here we present a systematic review and meta-analysis to define the proportion of EC patients who test positive for LS. This work includes data from 53 studies and 12,633 participants with EC who underwent IHC, MSI, methylation, or germline analysis to diagnose LS. From these data, of 100 unselected cases of EC, approximately 3 people are estimated to have LS, consistent with current literature.^76,77^ While this is a modest percentage of positive tests, each diagnosis allows for cascade testing of family members. It is estimated that 80% of living first-degree relatives will accept LS screening.^78^ Newly diagnosed LS patients are able to benefit from interventions to prevent the development of other LS cancers, most notably CRC. The results of this meta-analysis are summarized in supplementary figure S12 showing the estimated outcome from each stage of the LS diagnostic pathway.

Our results do not support the use of a particular tumor triage method. The proportion of positive test results if IHC or MSI was used as the initial tumor test (0.26 vs. 0.27 *p* value = 0.5 [Student's *t* test]) was similar. This small difference could be explained by the commonly used Bethesda panel for the detection of MSI, which has only been validated in CRC and not EC.^79^ In addition, MSH6 deficient EC can be microsatellite low or stable.^80^ That said, IHC does enable a more targeted application of *MLH1* promoter hypermethylation testing, given that it need only be applied to MLH1 deficient tumors. In addition, germline analysis could be limited to the gene(s) that corresponds to the protein lost; this has potential cost saving implications.

Preselecting EC populations by age or clinical criteria did not significantly change the proportion of positive IHC or MSI results, although we did find higher proportions in these subgroups. This is somewhat surprising, as preselected populations would be expected to harbor more women with LS. This may be partly explained by the later age of onset seen with *MSH6* pathological variants and truncating variants.^81^ In other words, the application of age cutoffs reduces specificity without a significant increase in sensitivity. However, universal testing does seem to decrease the yield of pathogenic variant carriers; this may arise from somatic events that lead to false positives at the tumor triage stage. *MLH1* methylation is associated with increased age and so is more common in older (unselected) populations.^82^ Subgroup analysis of studies that did not use a tumor triage stage, instead using direct germline sequencing, found a higher proportion of LS carriers (0.06). This could suggest that tumor triage itself misses potentially 50% of LS carriers. However, there were only three studies in the subgroup analysis. In addition, one of the studies preselected those who had germline testing on the basis of age (<50 years). However, this finding should encourage debate as to the application of NGS without tumor-based triage in EC populations; even more so given the decreasing cost of this technology.

Our work has several key strengths. Our conclusions are based on the results of over 50 studies and 12,600 participants; the search criteria were purposely broad as to capture the maximum number of studies. During the screening phase, three independent reviewers ensured the accuracy of study selection and data capture. Therefore, the foundations of our meta-analysis were robust. In addition, we have estimated the proportions of positive results seen in IHC, MSI, *MLH1* methylation, and germline testing with a high degree of precision, as reflected in the narrow 95% confidence intervals in our meta-analysis.

The heterogeneity across the studies included in our review was high, and limits the strength of our conclusions. This is a reflection of the varying quality and rigor of the included studies, some of which had small numbers of participants, and were subject to bias. The majority of studies used retrospective cohorts. Furthermore, many studies failed to complete the indicated testing in their cohorts, leading to ambiguity in their conclusions. This is evidenced by the lower proportion of LS pathological variants in those studies with complete germline analysis of Lynch-like tumors versus those with partial germline analysis (0.21 vs. 0.29). To allow for the pooling of such heterogeneous data, studies were grouped according to selection and diagnostic method. All studies that were grouped reported the same endpoint.

Another potential weakness of this study is the evolution in diagnostic technology over time. The included studies were published between 1996 and 2017. During this time diagnostic technology and guidelines have evolved significantly. Although IHC based diagnostics has remained relatively constant, MSI diagnostics has been informed by the adoption of the Bethesda panel in 1998 and the development of the more modern panels such as the pentaplex and hexaplex panels, which became widely applied to clinical practice after 2011.^83^ Even so, analysis of MSI testing results pre- vs. post-2011 did not find a significant difference (*t* test *p* value = 0.11). Only one study predates the Bethesda guidelines. The area of germline diagnostics remains innovative, but again analysis of pre- vs. post-2011 proportions of germline pathogenic variants found after tumor triage was not significant (*t* test *p* value = 0.14). Generalizability is limited by the predominance of North American and European populations in our study. Most took place in insurance-based health-care systems, which impact negatively on the uptake of genetic testing.^84^ Therefore, it could be that the proportions are an underestimate due to reduced uptake of testing, especially in high-risk groups such as the young or those with a strong family history.

In summary, ours is the first meta-analysis to examine the proportion of EC cases that are associated with LS. Different tumor triage methods did not affect estimates of the proportion of EC associated with LS, which remained constant at around 3%. Our findings suggest that a similar proportion of EC patients will test positive for LS as seen in CRC LS screening. This supports the move toward the introduction of universal screening for LS in EC.

## Supplementary information

Supplementary Materials
