# Supplementary material for: The proportion of endometrial cancers associated with Lynch syndrome: a systematic review of the literature and meta-analysis
Source: Genet Med. 2019 May 14;21(10):2167–80. doi: 10.1038/s41436-019-0536-8 (PMC8076013; doi:10.1038/s41436-019-0536-8)
Supplement: Supplementary file 1 — Supplementary Materials [file 41436_2019_536_MOESM1_ESM.docx]

The proportion of endometrial cancers associated with Lynch Syndrome: a systematic review of the literature and meta-analysis

SUPPLEMENTARY MATERIALS

Table of Contents

Appendix 1 – Excluded Studies 2

Table S1: Studies excluded with reasons after full manuscript review. 6

Appendix 2- Biases of included studies 7

Figure S1- Risk of bias and applicability concerns summary: review authors' judgements about each domain for each included study. Scores arise from the Newcastle-Ottawa Scale for observational studies ^28^. 7

Figure S2- Risk over basis represented as an overall proportion. 8

Appendix 3 – Overall gene distribution from germline sequencing 9

Figure S3: A graphic representation of the MMR pathogenic variants in women with endometrial cancer in the reported studies. 9

Appendix 4 – MLH1 methylation analysis 10

Figure S4: The incidence of MMR abnormality after the removal of *MLH1* results found to have hypermethylation 10

Figure S5: The incidence of MSI-H abnormality after the removal of *MLH1* results found to have hypermethylation 11

Appendix 5- Subgroup analysis 12

Figure S7: Subgroup analysis of immunohistochemistry proportions from studies undertaken in <50 years old populations 12

Figure S8: Subgroup analysis of microsatellite instability proportions from studies undertaken in non-western populations. 12

Figure S10: Subgroup analysis of pathogenic variants proportions from studies that undertook complete germline testing of all endometrial cancers suggestive of Lynch syndrome on the basis of their tumor triage. 13

Figure S11: Subgroup analysis of pathogenic variants proportions from studies that undertook germline analysis without a priori tumor based triage 13

REFERENCES 15

# Appendix 1 – Excluded Studies

| Title | Author | Date | Reason for exclusion |
| --- | --- | --- | --- |
| High frequency of microsatellite instability and loss of mismatch-repair protein expression in patients with double primary tumors of the endometrium and colorectum.^1^ | M Plank et al | 2002 | Population is of women with metachronous CRC and EC and therefore not reflective of a screening population |
| Identification of cancer patients with Lynch syndrome: clinically significant discordances and problems in tissue-based mismatch repair testing.^2^ | AN Bartley et al | 2012 | Comparison of tests taken from published data |
| Hereditary nonpolyposis colorectal cancer: a call for attention.^3^ | S Syngal | 2000 | Editorial |
| Universal tumor screening for Lynch syndrome: Assessment of the perspectives of patients with colorectal cancer regarding benefits and barriers.^4^ | JE Hunter et al | 2015 | CRC population. This paper focuses on the acceptability of testing |
| Loss of Mismatch Repair Protein Expression in Unselected Endometrial Adenocarcinoma Precursor Lesions.^5^ | KR Vierkoetter et al | 2016 | Not an EC population |
| Molecular epidemiological and mutational analysis of DNA mismatch repair (MMR) genes in endometrial cancer patients with HNPCC-associated familial predisposition to cancer.^6^ | Y Hirai et al | 2008 | Review with some primary data. Data extraction not possible due to limited information and no response from author |
| Prediction of a mismatch repair gene defect by microsatellite instability and immunohistochemical analysis in endometrial tumours from HNPCC patients.^7^ | WJ de Leeuw et al | 2000 | Population was known LS carriers |
| Identification of HNPCC by molecular analysis of colorectal and endometrial tumors.^8^ | HF Vassen et al | 2004 | Mostly CRC; limited information on EC cases included |
| Lynch Syndrome Screening in the Gynecologic Tract: Current State of the Art.^9^ | AM Mills et al | 2016 | Review |
| How should women with early-onset endometrial cancer be evaluated for lynch syndrome?^10^ | ND Kauff | 2007 | Editorial |
| Tumor Screening and DNA Testing in the Diagnosis of Lynch Syndrome.^11^ | L Usha et al | 2016 | Case report |
| Two Japanese kindreds occurring endometrial cancer meeting new clinical criteria for hereditarynon-polyposis colorectal cancer (HNPCC), Amsterdam Criteria II.^12^ | K Banno et al | 2004 | Case report |
| Utility of endometrial sampling prior to risk-reducing hysterectomy in a patient with Lynch syndrome.^13^ | MK Frey et al | 2016 | Case report |
| The Role of Expanded Testing for Lynch Syndrome in Women With Endometrial Cancer. ^13^ | MK Barton et al | 2011 | Modelling without actual real life data |
| Unusual Mismatch Repair Immunohistochemical Patterns in Endometrial Carcinoma.^15^ | JC Watkins et al | 2016 | Data published previously elsewhere |
| Using multi-gene testing to broaden the understanding of inherited endometrial cancer.^16^ | LE Panos et al | 2015 | LS not included in the diagnostic panel |
| Mutation spectrum and risk of colorectal cancer in African American families with Lynch syndrome.^17^ | RS Guindalini et al | 2015 | Population was known LS carriers |
| Correlation between body mass index and prevalence of hereditary nonpolyposis colorectalcancer in Korean patients with endometrial cancer.^18^ | HJ Yoo et al | 2012 | Non-incidence study |
| Clustering of Lynch syndrome malignancies with no evidence for a role of DNA mismatch repair.^19^ | AS Cases et al | 2008 | Non-incidence study |
| Clinicopathologic significance of DNA mismatch repair protein defects and endometrial cancer in women 40years of age and younger.^20^ | KK Shih et al | 2011 | Data extraction not possible due to limited information and no response from author |
| Outcomes of screening endometrial cancer patients for Lynch syndrome by patient-administered checklist.^21^ | MS Daniels et al | 2013 | Data extraction not possible due to limited information and no response from author |
| Screening for Lynch syndrome using risk assessment criteria in patients with ovarian cancer.^22^ | T Takashi et al | 2018 | Results were inconsistent |
| Identifying Lynch Syndrome in Women Presenting with Endometrial Carcinoma Under the Age of 50 Years.^23^ | A Anagnostopoulos et al | 2017 | Data extraction not possible due to limited information and no response from author |
| Clinicopathological significance of endometrial cancer with MSH2 deficiency.^24^ | J Haraga et al | 2017 | Data extraction not possible due to limited information and no response from author |
| Pathogenic and likely pathogenic variant prevalence among the first 10,000 patients referred for next-generation cancer panel testing.^25^ | LR Susswein et al | 2016 | Data extraction not possible due to limited information - specifically it was not possible to identify the methods and outcomes of tumour based triage |
| Prevalence of germline mutations of MLH1 and MSH2 in hereditary nonpolyposis colorectal cancer families from Spain.^26^ | T Caldes et al | 2002 | Data extraction not possible due to limited information and no response from author |
| Microsatellite instability and expression of MLH1 and MSH2 in normal and malignant endometrial and ovarian epithelium in hereditary nonpolyposis colorectal cancer family members.*^27^* | Y Ichikawa et al | 1999 | Less than 15 ECs |

## Table S1: Studies excluded with reasons after full manuscript review.

Abbreviations: EC: Endometrial Cancer; CRC: Colorectal cancer

# Appendix 2- Biases of included studies


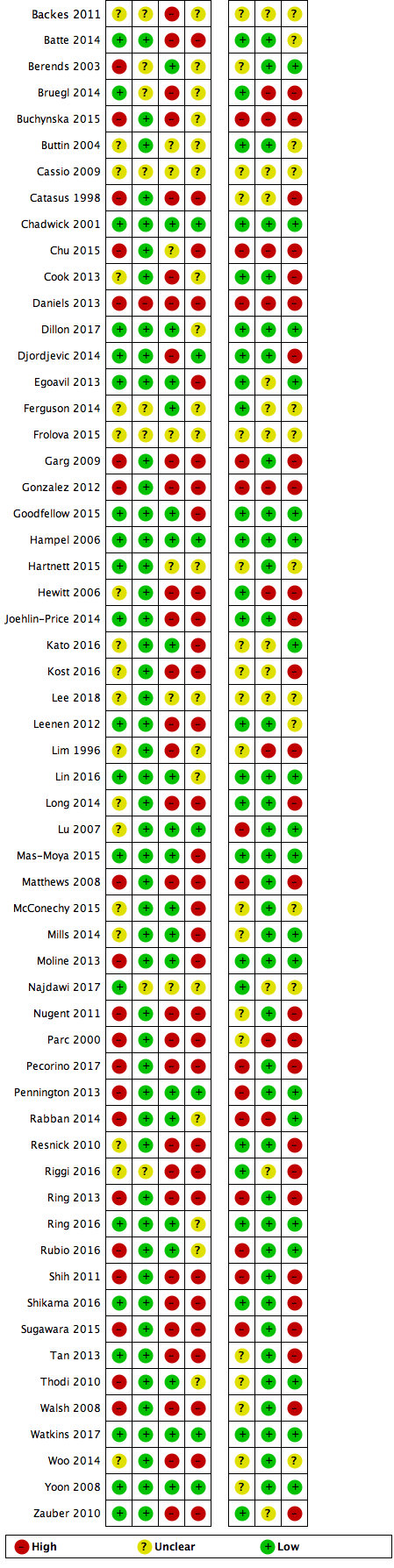

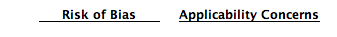


A

B a

C a

D a

E a

F a

G a

A: Selection

B: Comparability

C: Exposure

D: Outcome

E: Selection

F: Comparability

G: Outcome

##

## Figure S1- Risk of bias and applicability concerns summary: review authors' judgements about each domain for each included study. Scores arise from the Newcastle-Ottawa Scale for observational studies ^28^.

##


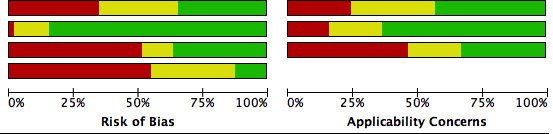

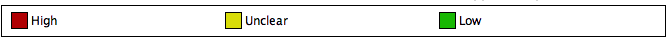


A

B

C

A

D

E

F

G

## Figure S2- Risk over basis represented as an overall proportion.

# Appendix 3 – Overall gene distribution from germline sequencing

#

## Figure S3: A graphic representation of the MMR pathogenic variants in women with endometrial cancer in the reported studies. Note EPCAM was included in MSH2 (n=2).

##

# Appendix 4 – *MLH1* methylation analysis


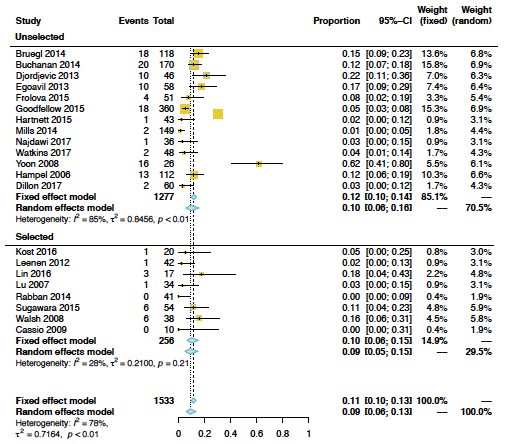


## Figure S4: The incidence of MMR abnormality after the removal of MLH1 results found to have hypermethylation

##
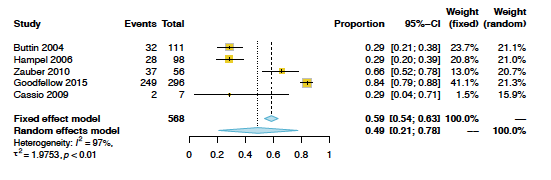


## Figure S5: The incidence of MSI-H abnormality after the removal of *MLH1* results found to have hypermethylation

# Appendix 5- Subgroup analysis


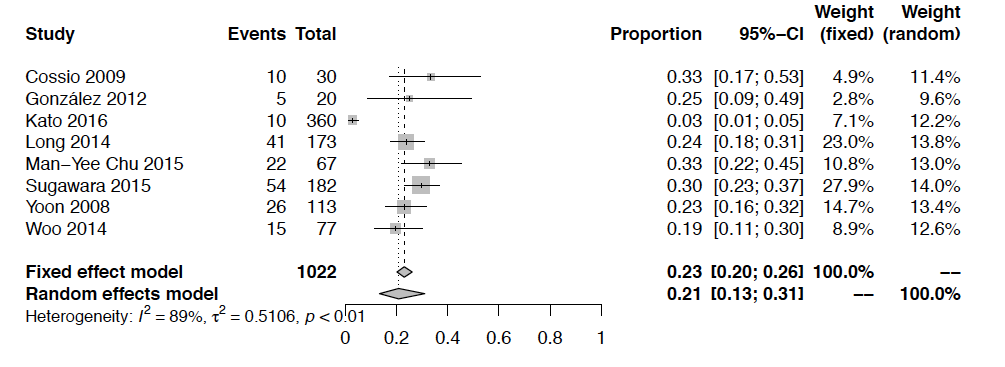


Figure S6: Subgroup analysis of immunohistochemistry proportions from studies undertaken in non-western populations.


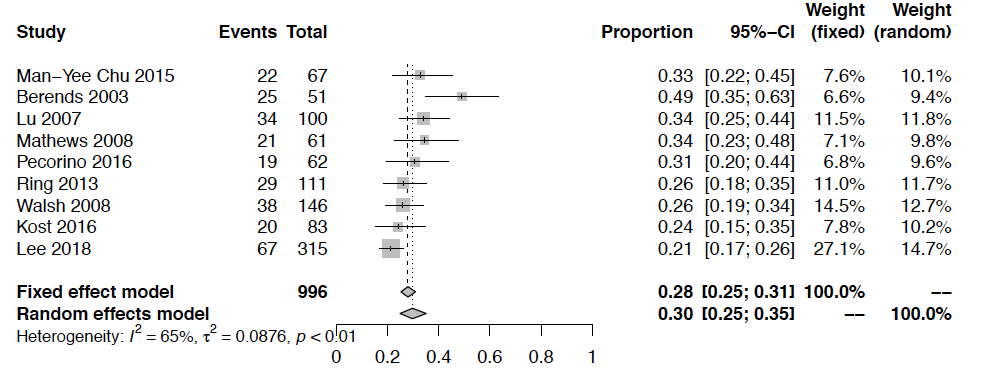


##
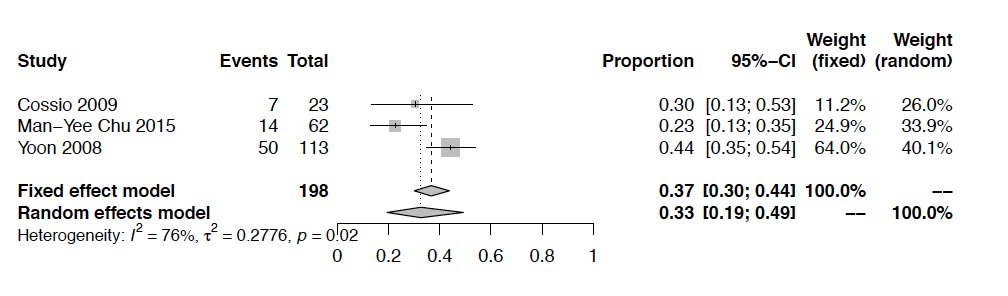
Figure S7: Subgroup analysis of immunohistochemistry proportions from studies undertaken in <50 years old populations

## Figure S8: Subgroup analysis of microsatellite instability proportions from studies undertaken in non-western populations.


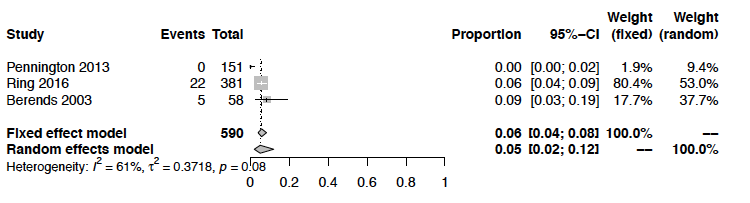


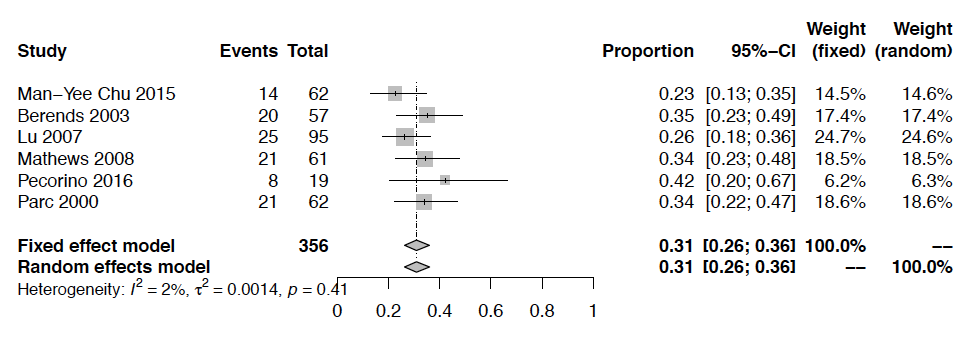
Figure S9: Subgroup analysis of microsatellite instability proportions from studies undertaken in <50 years old populations.

## Figure S10: Subgroup analysis of pathogenic variants proportions from studies that undertook complete germline testing of all endometrial cancers suggestive of Lynch syndrome on the basis of their tumor triage.

##
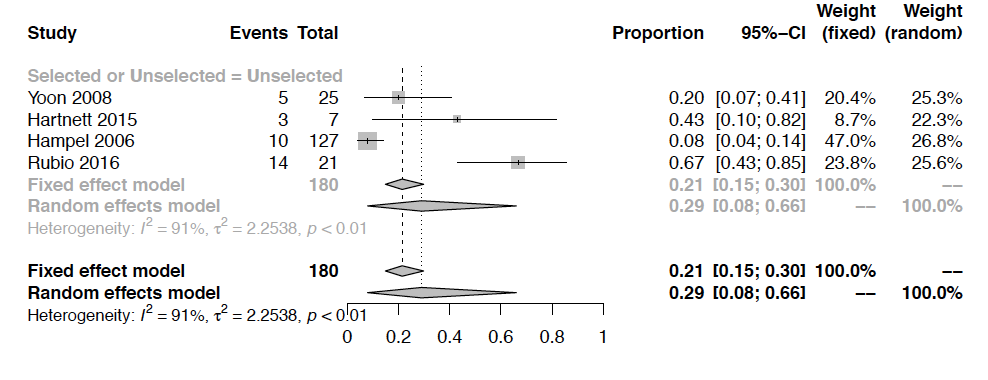


## Figure S11: Subgroup analysis of pathogenic variants proportions from studies that undertook germline analysis without a priori tumor based triage

Unselected Endometrial Cancer

MSI Analysis

(n=4,310)

MMR IHC Analysis

(n=10,460)

MLH1/PMS2 Loss

Methylation Testing

Normal

Methylated

Normal

MSI-H

Tumor Indicated Germline Analysis for Lynch syndrome

Normal

Pathogenic Variant

0.74 (74%)

0.27 (27%)

Normal

0.08 (8%)

0.18 (18%)

0.88 (16%)

0.12 (2%)

0.2-0.3 (2-3%)

Methylation Testing

Normal

Methylated

0.59 (16%)

0.41 (12%)

0.7-0.8 (97-98%)

0.74 (74%)

MSH2/MSH6 Loss

Figure S12: A composite flowchart of test positivity in unselected ECs based on the data within this meta-analysis. Each number is the proportion of samples that will go on to have the indicated outcome. Each step is sequential as is the corresponding proportion. Proportions for the MMR were drawn from studies that utilised *MLH1* methylation testing and described outcomes for all 4 proteins. The percentages in brackets indicate the percentage of positive results from an overall sample of unselected ECs. Abbreviations: MMR: Mismatch repair, IHC: immunohistochemistry, MSI- Microsatellite instability, MSI-H, Microsatellite instability-high

# **REFERENCES**

1. Planck M, Rambech E, Möslein G, Müller W, Olsson H, Nilbert M. High frequency of microsatellite instability and loss of mismatch‐repair protein expression in patients with double primary tumors of the endometrium and colorectum. *Cancer*. 2002;94(9):2502-2510. doi:10.1002/cncr.10501.

2. Bartley AN, Luthra R, Saraiya DS, Urbauer DL, Broaddus RR. Identification of cancer patients with Lynch syndrome: clinically significant discordances and problems in tissue-based mismatch repair testing. *Cancer Prev Res (Phila)*. 2012;5(2):320-327. doi:10.1158/1940-6207.CAPR-11-0288.

3. Syngal S. Hereditary nonpolyposis colorectal cancer: a call for attention. *JCO*. 2000;18(11):2189-2192. doi:10.1200/JCO.2000.18.11.2189.

4. Hunter JE, Zepp JM, Gilmore MJ, et al. Universal tumor screening for Lynch syndrome: Assessment of the perspectives of patients with colorectal cancer regarding benefits and barriers. *Cancer*. 2015;121(18):3281-3289. doi:10.1002/cncr.29470.

5. Vierkoetter KR, Kagami LAT, Ahn HJ, Shimizu DM, Terada KY. Loss of Mismatch Repair Protein Expression in Unselected Endometrial Adenocarcinoma Precursor Lesions. *Int J Gynecol Cancer*. 2016;26(2):228-232. doi:10.1097/IGC.0000000000000606.

6. Hirai Y, Banno K, Suzuki M, et al. Molecular epidemiological and mutational analysis of DNA mismatch repair (MMR) genes in endometrial cancer patients with HNPCC-associated familial predisposition to cancer. *Cancer Science*. 2008;99(9):1715-1719. doi:10.1111/j.1349-7006.2008.00886.x.

7. de Leeuw WJ, Dierssen J, Vasen HF, et al. Prediction of a mismatch repair gene defect by microsatellite instability and immunohistochemical analysis in endometrial tumours from HNPCC patients. *The Journal of Pathology*. 2000;192(3):328-335. doi:10.1002/1096-9896(2000)9999:9999<::AID-PATH701>3.0.CO;2-2.

8. Vasen HFA, Hendriks Y, de Jong AE, et al. Identification of HNPCC by Molecular Analysis of Colorectal and Endometrial Tumors. *Disease markers*. 2004;20(4-5):207-213. doi:10.1155/2004/391039.

9. Mills AM, Longacre TA. Lynch Syndrome Screening in the Gynecologic Tract: Current State of the Art. *The American Journal of Surgical Pathology*. 2016;40(4):e35-e44. doi:10.1097/PAS.0000000000000608.

10. Kauff ND. How should women with early-onset endometrial cancer be evaluated for lynch syndrome? *J Clin Oncol*. 2007;25(33):5143-5146. doi:10.1200/JCO.2007.13.4940.

11. Usha L, Dewdney SB, Buckingham LE. Tumor Screening and DNA Testing in the Diagnosis of Lynch Syndrome. *JAMA*. 2016;316(1):93-94. doi:10.1001/jama.2016.8286.

12. Banno K, Susumu N, Hirao T, et al. Two Japanese kindreds occurring endometrial cancer meeting new clinical criteria for hereditary non-polyposis colorectal cancer (HNPCC), Amsterdam Criteria II. *J Obstet Gynaecol Res*. 2004;30(4):287-292. doi:10.1111/j.1447-0756.2004.00195.x.

13. Frey MK, David-West G, Mittal KR, Muggia FM, Pothuri B. Utility of endometrial sampling prior to risk-reducing hysterectomy in a patient with Lynch syndrome. *Ecancermedicalscience*. 2016;10:613. doi:10.3332/ecancer.2016.613.

14. Barton MK. The role of expanded testing for Lynch syndrome in women with endometrial cancer. *CA: A Cancer Journal for Clinicians*. 2011;61(5):285-286. doi:10.3322/caac.20126.

15. Watkins JC, Nucci MR, Ritterhouse LL, Howitt BE, Sholl LM. Unusual Mismatch Repair Immunohistochemical Patterns in Endometrial Carcinoma. *The American Journal of Surgical Pathology*. 2016;40(7):909-916. doi:10.1097/PAS.0000000000000663.

16. Panos LE, Chao E, McFarland R, LaDuca H. Using multi-gene testing to broaden the understanding of inherited endometrial cancer. *Gynecol Oncol*. 2015;137:19-20. doi:10.1016/j.ygyno.2015.01.046.

17. Guindalini RSC, Win AK, Gulden C, et al. Mutation Spectrum and Risk of Colorectal Cancer in African American Families with Lynch Syndrome. *Gastroenterology*. 2015;149(6):1446-1453. doi:10.1053/j.gastro.2015.07.052.

18. Yoo HJ, Joo J, Seo SS, et al. Correlation between body mass index and prevalence of hereditary nonpolyposis colorectal cancer in Korean patients with endometrial cancer. *International Journal of Gynecological Cancer*. 2012;22(2):267-272. doi:10.1097/IGC.0b013e31823b3650.

19. Case AS, Zighelboim I, Mutch DG, et al. Clustering of Lynch syndrome malignancies with no evidence for a role of DNA mismatch repair. *Gynecol Oncol*. 2008;108(2):438-444. doi:10.1016/j.ygyno.2007.09.036.

20. Shih KK, Garg K, Levine DA, et al. Clinicopathologic significance of DNA mismatch repair protein defects and endometrial cancer in women 40years of age and younger. *Gynecol Oncol*. 2011;123(1):88-94. doi:10.1016/j.ygyno.2011.06.005.

21. Daniels MS, Urbauer DL, Zangeneh A, Batte BAL, Dempsey KM, Lu KH. Outcomes of screening endometrial cancer patients for Lynch syndrome by patient-administered checklist. *Gynecol Oncol*. 2013;131(3):619-623. doi:10.1016/j.ygyno.2013.10.011.

22. Takeda T, Tsuji K, Banno K, et al. Screening for Lynch syndrome using risk assessment criteria in patients with ovarian cancer. *J Gynecol Oncol*. 2018;29(3):e29. doi:10.3802/jgo.2018.29.e29.

23. Anagnostopoulos A, McKay VH, Cooper I, Campbell F, Greenhalgh L, Kirwan J. Identifying Lynch Syndrome in Women Presenting With Endometrial Carcinoma Under the Age of 50 Years. *International Journal of Gynecological Cancer*. 2017;27(5):931-937. doi:10.1097/IGC.0000000000000962.

24. Haraga J, Nagasaka T, Nakamura K, et al. Abstract 4277: Clinicopathological significance of endometrial cancer with MSH2 deficiency. *Cancer Res*. 2017;77(13 Supplement):4277-4277. doi:10.1158/1538-7445.AM2017-4277.

25. Susswein LR, Marshall ML, Nusbaum R, et al. Pathogenic and likely pathogenic variant prevalence among the first 10,000 patients referred for next-generation cancer panel testing. *Genet Med*. 2015;18(8):823-832. doi:10.1038/gim.2015.166.

26. Caldés T, Godino J, la Hoya de M, et al. Prevalence of germline mutations of MLH1 and MSH2 in hereditary nonpolyposis colorectal cancer families from Spain. *Int J Cancer*. 2002;98(5):774-779.

27. Ichikawa Y, Lemon SJ, Wang S, et al. Microsatellite Instability and Expression of MLH1 and MSH2 in Normal and Malignant Endometrial and Ovarian Epithelium in Hereditary Nonpolyposis Colorectal Cancer Family Members. *Cancer Genetics and Cytogenetics*. 1999;112(1):2-8. doi:10.1016/S0165-4608(98)00252-0.

28. Stang A. Critical evaluation of the Newcastle-Ottawa scale for the assessment of the quality of nonrandomized studies in meta-analyses. *Eur J Epidemiol*. 2010;25(9):603-605. doi:10.1007/s10654-010-9491-z.
